# Secondary stone formation 8 weeks after percutaneous nephrolithotomy treatment: A case report

**DOI:** 10.1097/MD.0000000000026091

**Published:** 2021-05-28

**Authors:** Qiong Deng, Hongliang Wang, Yulin Lai, Hui Liang

**Affiliations:** a Department of Urology, Affiliated Longhua People's Hospital; b College of Basic Medicine, Southern Medical University, Shenzhen, Guandong, China.

**Keywords:** 16S RNA sequencing, percutaneous nephrolithotomy, renal calculi, renal pus

## Abstract

**Introduction:**

This work reports a patient with recurrent renal calculi subjected to three surgeries in half a year to be in the same position, and the high-throughput sequencing data showed different species in the renal pus and urine samples, which suggested that partial renal infection or stone formation can be judged by the bacteria in urine.

**Patient concerns:**

The female patient aged 43 years was referred to the authors’ department on April 13, 2020, due to left waist pain and fever for 3 days.

**Diagnosis:**

Kidney stones and hydronephrosis were determined by a urinary system computed tomography scan.

**Interventions:**

On April 20, 2020 and June 15, 2020, the patient was successfully treated with left percutaneous nephrolithotomy twice under general anesthesia. An investigation on the health and eating habits of the patient within 6 months was completed at the last admission. The components of the second renal calculus sample were analyzed with an infrared spectrum analyzer. The third renal stone (renal pus, triplicates) was subjected to microbial metagenome sequencing, and urine samples before and after surgery were subjected to 16S RNA sequencing by SEQHEALTH (Wuhan, China).

**Outcomes:**

After percutaneous nephrolithotomy, the left kidney stones were basically cleared, stone analysis revealed that the main components were calcium oxalate monohydrate, silica, and a small amount of calcium oxalate dehydrate. Although the urine samples exhibited differences, the renal pus and urine sample shared a single species.

**Conclusion:**

It is not clear that the prospects of partial renal infection or stone formation can be judged by the bacteria in urine.

## 1 Introduction

Renal calculi, also known as nephrolithiasis or urolithiasis, have a prevalence of 1% to 15%, and their incidence is reported to be increasing worldwide.^[1,2]^ The development of kidney stones as a consequence of the exposure of renal epithelial cells to oxalate and calcium oxalate crystals can cause the blockage of the ureter, blood in the urine, vomiting, or painful urination, resulting in damage to the kidneys.^[3]^ Once afflicted, renal calculi tend to be recurrent at a rate of up to 50% in the majority of cases within 5 years after the first stone event.^[4]^

## 2 Case presentation

**Figure 1:**
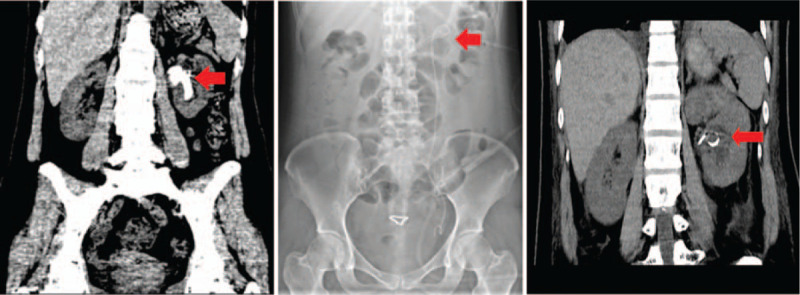
Imaging data of the patient's first hospitalization in the authors’ department. Left panel, the computed tomography (CT) scan showed multiple stones in the left kidney and hydronephrosis after admission on April 13, 2020. Postoperative reexamination of the plain abdominal radiograph (Middle panel) and CT scan (Right Panel) revealed that the left kidney stones were basically cleared on April 22, 2020.

A 43-year-old woman was referred to the authors’ department on April 13, 2020, due to left waist pain and fever for 3 days. The patient underwent extracorporeal shock wave lithotripsy treatment for left kidney stones >10 years ago, and left ureteroscopic lithotripsy (URL) 4 months before her visit. After admission, the patient underwent a urinary system computed tomography (CT) scan, which indicated multiple stones in the left kidney and hydronephrosis. The maximum cross-sectional size of the stones was about 13 × 32 mm (Fig. 1, left). On April 20, 2020, the patient was successfully treated with left percutaneous nephrolithotomy under general anesthesia. Postoperative reexamination of the plain abdominal radiograph and CT scan revealed that the left kidney stones were basically cleared (Fig. 1, middle and right panels). Stone analysis revealed that the main components were calcium oxalate monohydrate, silica, and a small amount of calcium oxalate dehydrates.

**Figure 2:**
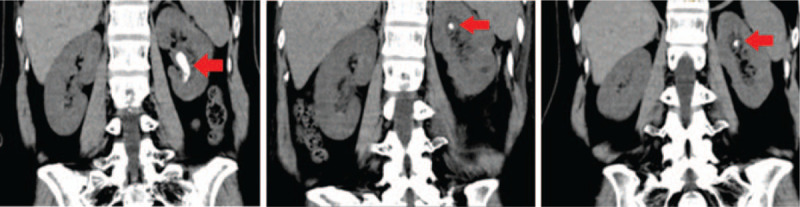
The second percutaneous nephrolithotomy computed tomography (CT) images and postoperative reexamination results. Left panel, the CT scan of the urinary system on June 10, 2020 revealed that there were more left renal calyx stones than before (April 13, 2020). Middle panel postoperative examination of the CT scan of the urinary system revealed that the left kidney stones were significantly reduced on June 16, 2020. Right panel, on August 23, 2020, the CT scan shows that there was no significant change in the residual stones with the middle panel.

The patient returned to the hospital on June 10, 2020, for the removal of the ureteral stent, but a reexamination of the CT scan of the urinary system revealed that there were more left renal calyx stones than before (Fig. 2, left). Considering that the left kidney stones were infectious stones, they had relapsed in the short term. On June 15, 2020, left percutaneous nephrolithotomy was performed again under general anesthesia. Postoperative examination of the CT scan of the urinary system revealed that the left kidney stones were significantly reduced (Fig. 2, middle). The patient was discharged smoothly. A CT scan of the urinary system was examined again on August 23, 2020, and it was found that there was no significant change in the residual stones in the left kidney (Fig. 2, right).

## 3 Discussions

It is rare for a patient subjected to 3 surgeries in half a year to be in the same position. The patient was 43 years’ old with a BMI of 21.5 and confirmed *Helicobacter pylori* infection. The patient self-reported having no alcoholic beverage intake, a stable mood, a regular diet, low- intensity work, and sufficient rest, but less exercise and less daily water consumption (1000–1500 mL/day).

**Figure 3:**
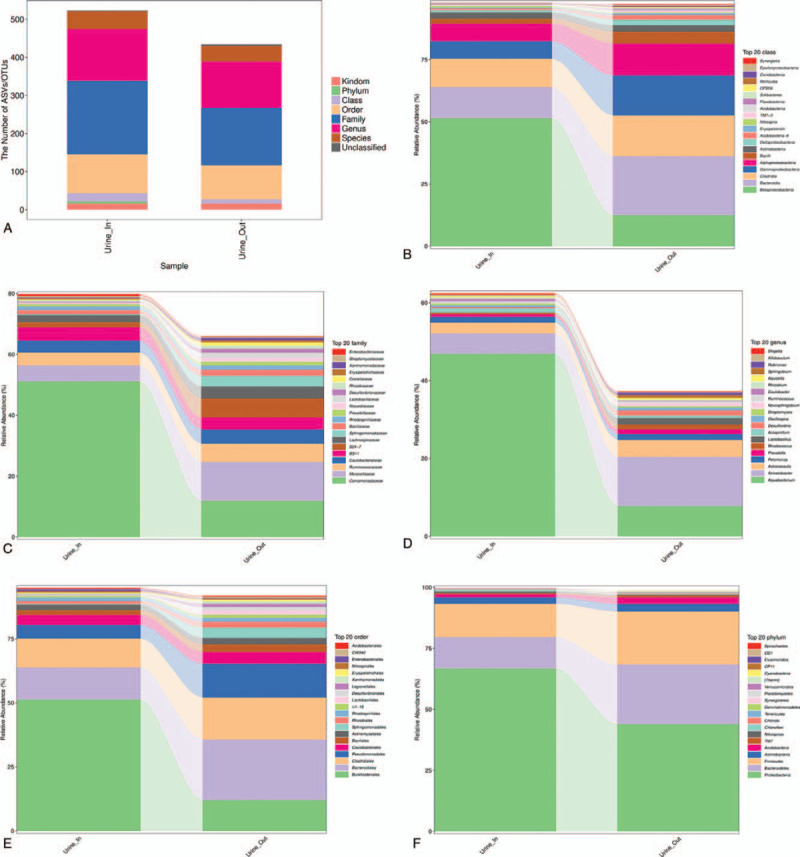
16S RNA sequencing results of the urine samples. (A) The numbers of ASVs/OTUs in the samples. The relative abundance of the classes (B), families (C), genera (D), top 20 orders (E), top 20 phyla (F) were presented.

A portion of the 16S RNA sequencing data is presented in Figure 3. The urine samples revealed differences in the numbers of ASVs/OTUs (Fig. 3A). Regarding the classes (Fig. 3B), the relative abundance of Betaproteobacteria appeared to be higher in the Urine_In group (urine collected at check-in, June 11, 2020), whereas the Urine_Out group (urine collected post-hospitalization, June 25, 2020) had higher percentages of Bacteroidia, Clostridia, Gammaproteobacteria, Alphaproteobacteria, and so on. It has been demonstrated that drinking water contains a high diversity of Betaproteobacteria, whose presence may not be innocuous. Betaproteobacteria are ubiquitous in different habitats, and have the potential to resist antibiotics either due to intrinsic or acquired mechanisms, and possess different virulence factors.^[5]^ The increase of Bacteroidia and Clostridia in the Urine_Out group agreed with the results of Kikuchi's study, in which normal control rats were compared with rats with chronic kidney disease.^[6]^ Regarding the families (Fig. 3C), more *Moraxellaceae*, *S24-7*, and *Sphingomonadaceae,* but less *Comamonadacear*, were found in the Urine_Out group. The presence of *Moraxellaceae* is higher in patients with end-stage renal disease than in control patients, which suggests that it may be used as a negative marker.^[7]^ Bacteria belonging to the taxa *Oxalobacteraceae*, *Bifidobacterium*, *Clostridium*, *S24-7*, *Lactobacillus*, and *Clostridiales* are known to harbor oxalate-degrading genes.^[8,9]^ Regarding the genera (Fig. 3D), more *Auqabacterium* was found in the Urine_In group, and more Acinetobacter was found in the Urine_Out group. Regarding the top 20 orders (Fig. 3E), the Urine_Out group had more Bacteroidales, Clostridiales, Pseudomonadales, and Sphingomonadales, and half of the orders in the Urine_In group was Burkholderiales. It has been reported that mice fed with high-salt diets, which induce gut dysbiosis and renal dysfunction, have an enriched abundance of Burkholderiales.^[10]^ Regarding the top 20 phyla (Fig. 3F), around two-thirds in the Urine_In group were Proteobacteria, and there were more Bacteroidetes and Firmicutes in the Urine_Out group. These data are consistent with a study that found that the proportions of Bacteroidetes and Firmicutes were significantly higher in the healthy controls than in urolithiasis patients^[11]^; the bacterial flora in the urine of the patients had changed significantly after treatment. This may have been due to the use of antibiotics, or it may have been because the microflora tended to be more stable.

**Figure 4:**
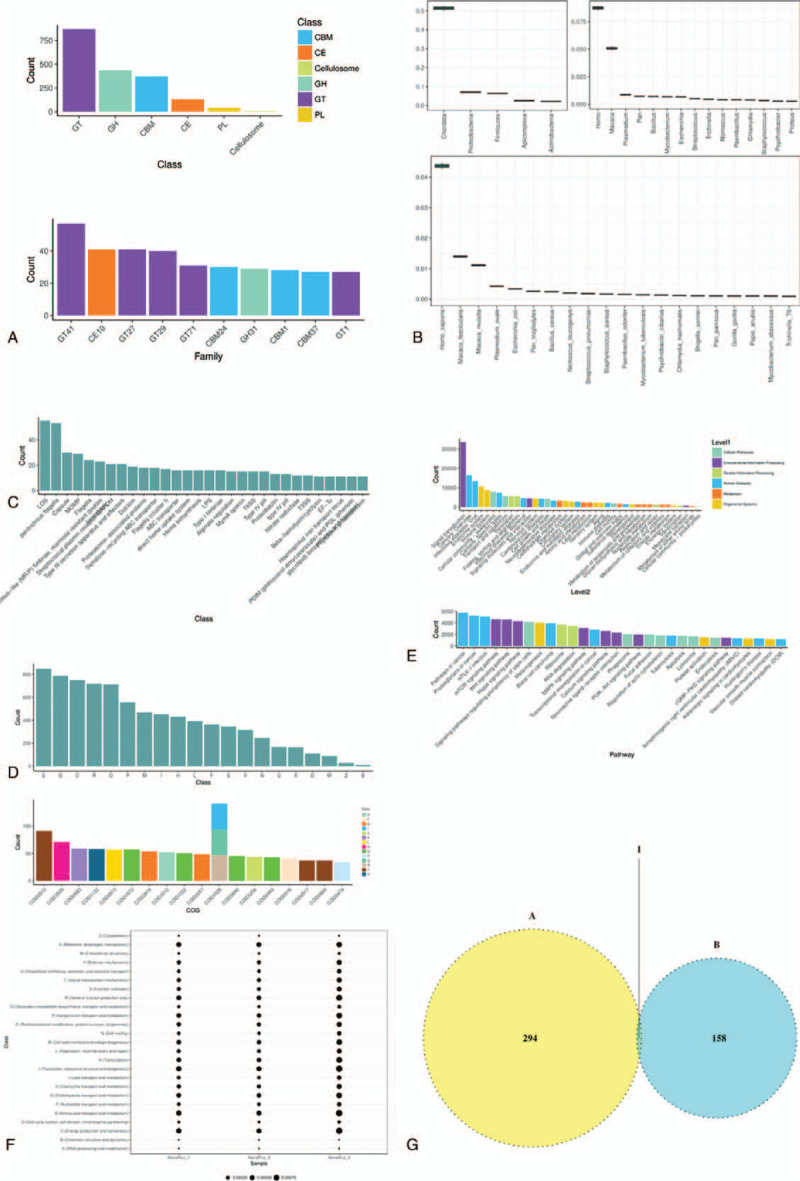
Microbial metagenome sequencing data of renal pus. (A) GT, GH, and CBM were the dominant classes and families in the renal pus. (B) Chordata, *Homo sapiens*, *Macaca tascicularis*, and *Macaca mulatta* were the dominant species. (C) The prediction of the detected virulence factors by Virulence Factors Database DB. (D and F) The predicted proteins from triplicates were annotated, classified, and analyzed for protein evolution. (E) The gene set with the KEGG gene database was compared using DIAMOND. (G) The renal pus and urine samples shared 1 species.

Microbial metagenome sequencing was conducted on the renal pus samples collected at June 15, 2020 during the surgery, and the data are presented in Figure 4, as is the joint analysis with the 16S RNA results. Figure 4A shows that GT, GH, and CBM were the dominant classes and families in the renal pus. Chordata occupied half of the abundance, and *Homo sapiens*, *Macaca tascicularis*, and *Macaca mulatta* were the dominant species (Fig. 4B). As exhibited in Figure 4C, the Virulence Factors Database was used to predict the composition, structure, function, pathogenic mechanism, Virulence Island, sequence, and genomic information of the detected virulence factors. The Clusters of Orthologous Groups of proteins database was utilized, and the predicted proteins from triplicates were annotated, classified, and analyzed for protein evolution (Fig. 4D and F). As presented in Figure 4E, the Kyoto Encyclopedia of Genes and Genomes (KEGG) orthology (KO) annotation information from the KEGG database was obtained using DIAMOND to compare the gene set with the KEGG gene database (genes). The data suggested that 294 different types of microorganisms were found in the renal pus, and 158 species were found in the urine. However, surprisingly, the 2 types of samples share only 1 species (Fig. 4G). Previous studies have demonstrated that the outcomes of urine and renal stones are not always identical^[12–14]^; for example, in an American single-center study, *Staphylococcus* was identified as the most common bacteria in both urine and stones, whereas the bacterial spectrum of urine was not the same as that of stones.^[15]^

Judging from the results of this case, patients with recurrent stones should pay more attention to anti infection treatment, it is not clear that the prospects of partial renal infection or stone formation can be judged by the bacteria in urine.

## Author contributions

Q Deng analyzed and interpreted the patient data, prepared the manuscript. HL Wang was the physician in charge of the patient. YL Lai was a major contributor in revising the manuscript and an assistant in the surgery. H Liang was the chief physician and conceived the study. All authors read and approved the final manuscript.

**Data curation:** Yulin Lai.

**Investigation:** Qiong deng.

**Methodology:** Hongliang Wang.

**Supervision:** Hui Liang.

**Writing – original draft:** Qiong Deng, Yulin Lai.

**Writing – review & editing:** Hui Liang.
